# Model-based economic evaluation of non-pharmacological interventions for fatigue in patients with long-term medical conditions in the UK

**DOI:** 10.1136/bmjopen-2025-102846

**Published:** 2026-04-24

**Authors:** Mon Mon-Yee, Christopher Burton, Joanna Leaviss, Jessica E Forsyth, George Daly, Sarah Davis

**Affiliations:** 1 School of Medicine and Population Health, The University of Sheffield, Sheffield, UK

**Keywords:** Fatigue, Chronic Disease, HEALTH ECONOMICS

## Abstract

**Abstract:**

**Background:**

Persistent fatigue is a frequent symptom in chronic medical conditions. Systematic reviews of non-pharmacological interventions for fatigue have identified interventions that are effective at reducing fatigue, but there is limited published evidence on the cost-effectiveness of these interventions.

**Objective:**

To identify non-pharmacological fatigue interventions that have the potential to be cost-effective in patients with long-term medical conditions.

**Design:**

Decision analytic modelling with intervention costs estimated from staff time and quality-of-life outcomes mapped from a systematic review and network meta-analysis of fatigue outcomes.

**Setting:**

UK National Health Service (NHS).

**Participants:**

People with persistent fatigue associated with a chronic medical condition.

**Interventions:**

Non-pharmacological fatigue interventions versus usual care.

**Primary and secondary outcome measures:**

Net monetary benefit from a UK NHS and Personal Social Services perspective; quality-adjusted life years (QALYs) gained; intervention costs valued at 2022/23 prices; costs and benefits discounted at 3.5% per annum.

**Results:**

In the base-case analysis, expected costs from the probabilistic analysis for individual and group interventions were: £267 and £157 for physical activity promotion, £810 and £485 for cognitive behavioural therapy (CBT)-Fatigue and £462 and £214 for mindfulness. The expected QALYs gained were similar for mindfulness and physical activity promotion (0.061 and 0.060, respectively), but lower for CBT-Fatigue (0.045). All interventions provided positive incremental net monetary benefit (INMB) versus usual care when valuing a QALY at £20 000. However, since group interventions are less costly than individual ones, and we assumed equivalent clinical benefit, they are expected to provide greater INMB. These findings remained robust across different scenarios, except for CBT-Fatigue (individual), which had negative INMB in some scenarios.

**Conclusions:**

There remains uncertainty regarding which intervention is most cost-effective due to limitations in the underlying evidence base. Future research is recommended to compare the cost-effectiveness of these interventions across a broad population with different chronic conditions.

**STRENGTHS AND LIMITATIONS OF THIS STUDY**

- Fatigue outcomes were estimated from a robust systematic review and network meta-analysis that pooled data across studies conducted across multiple chronic conditions, with multiple sclerosis being most common.
- The treatment effects for interventions delivered to groups and individuals are assumed to be similar, but this assumption was only supported by an analysis exploring separate treatment effects for group and individual cognitive–behavioural therapy-Fatigue interventions.
- The results for group physical activity promotion and individual mindfulness interventions should be treated with caution owing to there only being data beyond end of treatment for individual physical activity promotion and group mindfulness interventions.
- Healthcare costs were restricted to staffing costs for delivering the intervention and therefore do not capture any impact on resource use outside of the intervention or any non-staff intervention costs such as access to a specific digital tool.

## Introduction

 Persistent fatigue is common in long-term medical conditions.^1^ While estimates vary, approximately half of patients with a long-term medical condition experience clinically significant fatigue.^2^ Fatigue affects people with a wide range of conditions, including rheumatoid arthritis, multiple sclerosis, chronic kidney disease and type 2 diabetes.^3^,^6^ Transdiagnostic factors account for considerably more of the variance in fatigue than the medical conditions themselves, suggesting important common risk factors and mechanistic pathways.^7^ The experience of fatigue also appears to show similarities across different conditions, particularly in the perceived nature and patterns of fatigue: often different to ordinary tiredness with an overwhelming intensity, feeling as though it would never go away and being unpredictable.^8^ Fatigue is often overlooked and can persist even after the underlying disease has been fully controlled.^8^ In addition to having an impact on quality of life,^9^ fatigue in long-term medical conditions is significantly associated with higher healthcare resource use, increased medical costs, more sick leave and reduced work productivity and daily functioning.^510^,^13^

Currently, there are no licensed pharmacological treatments for fatigue in chronic conditions. Several non-pharmacological interventions have been developed in order to address fatigue in such conditions, targeting physical or psychological aspects or a combination of both. A recent systematic review and network meta-analysis (NMA) assessed the clinical effectiveness of non-pharmacological interventions across a broad set of long-term conditions.^14^ While relatively few studies examined the effectiveness of interventions >3 months after the end of treatment, those that did suggested that interventions which promoted physical activity or were derived from cognitive–behavioural therapy (CBT) appeared likely to have clinically meaningful benefit. These benefits appeared comparable across the medical conditions studied.^14 15^ Despite increasing interest in non-pharmacological interventions, no previous study has evaluated the cost-effectiveness of such interventions across multiple conditions using decision analytic modelling.^14^

The aim of this research was to explore the potential cost-effectiveness of non-pharmacological interventions for fatigue in patients with long-term medical conditions in the UK.

## Methods

This work was conducted as a part of an evidence synthesis examining both the clinical and cost-effectiveness of non-pharmacological interventions for fatigue in long-term medical conditions.^14 15^ The review of clinical effectiveness studies including the NMA and the review of cost-effectiveness studies are reported elsewhere.^14 16^ Eligibility criteria for these reviews used the National Health Service (NHS) definition of long-term conditions as “an illness that cannot be cured but that can usually be controlled with medicines or other treatments”. The following conditions were explicitly excluded in the commissioning brief: ‘fatigue in people with cancer, in relation to or following from infection (HIV, Hepatitis C, Long Covid and ME/CFS) or resulting from injuries or developmental disorders’. It also excluded conditions in which symptoms, rather than observable pathology, were the defining features (eg, fibromyalgia or irritable bowel syndrome).

The NMA of randomised controlled trials (RCTs) synthesised outcomes across all conditions and applied a transdiagnostic assumption. The fatigue outcomes were assessed at three time points: end of treatment (EOT), short-term (ST), which was defined as up to 3 months after EOT and long-term (LT) defined as >3 months after EOT. There was a large diversity of non-pharmacological interventions reported across the RCTs included in the clinical effectiveness review. These were grouped into intervention categories to allow evidence to be pooled across studies, which were considered to have interventions that were sufficiently similar. A large number of interventions were identified and included in the NMA, and many of these failed to demonstrate treatment effects that persisted beyond EOT. We therefore chose to focus the de novo economic analysis on those interventions that had the strongest evidence for clinical effectiveness. Consequently, we selected interventions for inclusion in the de novo economic evaluation, which reported fatigue outcomes at LT follow-up, and which demonstrated a statistically significant difference in treatment effect compared with usual care in the NMA for either the ST or LT follow-up. In addition, interventions that were considered by our clinical experts to address mechanisms specific to a single condition (eg, breath control exercises in chronic obstructive pulmonary disease) and therefore not generalisable across different chronic conditions were excluded. The expected health outcomes and expected costs were estimated relative to usual care in order to align with the approach taken in the clinical effectiveness review and NMA. Any pharmacological or non-pharmacological treatment for the underlying condition that was not specifically intended to manage fatigue was assumed to be unaffected by whether the patient receives a non-pharmacological fatigue intervention or usual care. The cost-effectiveness analyses were conducted from the perspective of UK NHS and personal social services. Costs are reported in UK pound sterling, valued at 2022/23 prices and costs and benefits were discounted at 3.5% per annum. The data sources and assumptions included in the economic model were informed by discussion with clinical experts and patient and public involvement experts.

### Patient and public involvement

The research group included two members with relevant lived experience and an academic expert in patient and public involvement who were involved in discussions regarding the data sources and assumptions for the economic modelling. Their contributions were informed by five focus groups convened of people with fatigue associated with LT conditions.

### Health outcome measures

Only a minority of studies included in the NMA reported a measure of health utility directly. Therefore, we applied a mapping algorithm to estimate utility values from the fatigue outcomes estimated by the NMA for each intervention category.

#### Identification and selection of mapping algorithms

A targeted literature review was conducted to identify papers that describe statistical mapping methods to convert fatigue-specific patient-reported outcome measures used in chronic health conditions to health state utility values derived from generic preference-based measures (PBM), such as the Euroqol 5-Dimensions (EQ-5D) and the Short Form 6 Dimensions (SF-6D). The detailed methods are provided in the online supplemental appendix.

The searches were performed in four electronic databases (MEDLINE, PubMed, Web of Science and the Cumulative Index to Nursing and Allied Health Literature). Details are provided in the online supplemental appendix. Our electronic database searches identified 96 papers. A study by Goodwin *et al*^17^ that mapped from the Fatigue Severity Scale (FSS) to health state utility values using EQ-5D-3L, SF-6D and Multiple Sclerosis Impact Scale 8-Dimensions (MSIS-8D) was identified from both electronic database and the Health Economics Research Centre database searches. After removing duplicates and reviewing the papers, two additional studies^18 19^ were found mapping Checklist Individual Strength-F to EQ-5D-5L and fatigue measured on a visual analogue scale to EQ-5D-3L, respectively. These three papers are summarised in the online supplemental table S1. No further relevant studies were identified from our citation search. The studies by Eriksson *et al*^18^ and Bloem *et al*^19^ were considered less appropriate than the study by Goodwin *et al* because they included other patient characteristics (eg, disease severity measures, comorbidities, depression and anxiety scores) in their regressions, which were not consistently available in the studies included in our evidence synthesis. The study by Goodwin *et al* was therefore selected in preference to these studies. Goodwin *et al* used five regression models to map from either FSS total score or FSS item score to each PBM (EQ-5D-3L, SF-6D and MSIS-8D) using both ordinary least squares and censored least adjusted deviation specifications. The regression model mapping from total FSS to SF-6D was reported as performing better than the regression model mapping to EQ-5D, with SF-6D root mean square errors (RMSEs) of 0.10, compared with EQ-5D RMSEs of 0.257. The MSIS-8D mapping algorithm was considered less useful for estimating utilities in populations with chronic conditions other than multiple sclerosis. Although the algorithm mapping to SF-6D was derived in a multiple sclerosis population, we felt this was broadly applicable to other populations of patients with chronic conditions as both the FSS and the SF-6D are generic tools that have been validated for use across a range of healthcare conditions.^20^ After considering the mapping studies identified in this review, we chose to use the regression model mapping from FSS to SF-6D provided by Goodwin *et al* in our de novo economic model.

#### Quality-adjusted life years

The QALY gains for each intervention and usual care were estimated using an area under the curve approach based on the average time points for fatigue outcomes reported in the studies contributing to the NMA. As the absolute utility values were not reported consistently across studies, we have mapped utility data from fatigue outcomes reported in the NMA. As studies included in the NMA reported outcomes using a range of fatigue scales, fatigue outcomes were pooled in the NMA on the standardised mean difference (SMD) scale. The treatment effects, expressed as the SMDs of the change in fatigue outcomes from baseline, were then converted to differences in FSS scores and mapped to SF-6D values to estimate the QALYs gained relative to the usual care. The choice to convert the treatment effects from SMDs to FSS was pragmatic and driven by the availability of an algorithm to map from FSS to health state utility values. Further details on the characteristics of the studies informing the NMA and the methods employed in the NMA can be found in the paper reporting the NMA.^14^

For each intervention category, the studies contributing to that intervention category in the NMA were used to estimate the timing for EOT and the two follow-up points (ST and LT) used when estimating QALYs. For studies that have reported fatigue scores for two different LT follow-ups, the base-case NMA included the longest follow-up data but a sensitivity analysis was conducted incorporating the earliest LT follow-up point (ie, closest to 3 months after EOT). This was done to ensure that any waning of treatment effect over time did not bias the analysis against studies that had a greater duration of follow-up. We explored the impact of using the data from this NMA sensitivity analysis in the economic analysis (scenario analysis) and adjusted the timing of the LT follow-up accordingly. The details of this NMA scenario analysis can be found in online supplemental table S9.

It was assumed that the baseline FSS score would be the same across interventions and the usual care comparator arm and this was based on an estimate from Goodwin *et al*^17^ (43.73, 95% CI 14.13 to 73.33). The absolute FSS score for usual care was kept fixed at its baseline value for all follow-up time points. For the intervention arms, the SMDs of fatigue scores provided by the NMA across all conditions for each intervention relative to the usual care were converted to a mean difference on the FSS scale using the SD of baseline FSS score of 15.1 reported by Goodwin *et al* in the base case. The SD of FSS scores derived from studies included within the NMA was used in the scenario analysis (see online supplemental appendix for further details). This difference in fatigue scores between intervention and usual care was used to estimate the absolute FSS score after baseline for each intervention at each follow-up point (absolute FSS score=difference in FSS score+baseline FSS score). It did not seem reasonable to assume a sudden return to baseline fatigue scores after the last follow-up. Therefore, it was assumed that interventions would experience a linear decline in treatment effect between the last follow-up and 24 months after baseline in the base case. The choice of a 24-month time horizon in the base case was informed by LT follow-up data from two studies, which showed the potential for treatment effects to be maintained beyond 1 year after EOT.^21 22^ As the durations of most interventions included in the economic model were <12 months, extending the time horizon to 24 months was considered reasonable to capture differences in cost-effectiveness across interventions. No further treatment effect was assumed to persist beyond that point. Scenarios exploring pessimistic (15-month time horizon) and optimistic (48-month time horizon) assumptions about the persistence of treatment effects were explored based on clinical expert advice. Additionally, a scenario assessed the assumption that FSS scores return immediately to baseline after the last follow-up. If data were missing at the ST follow-up point, a linear change in FSS between the EOT and LT follow-up points was assumed. This assumption was tested in the scenario analysis, where the treatment effect was assumed to be zero when data were unavailable. QALYs were discounted using a discount rate of 3.5% per annum, as recommended by the National Institute for Health and Care Excellence.^23^ Different discount rates of 1.5% and 6% were tested in the scenario analyses.

### Resource use and costs

The studies included in the NMA were examined to determine the resources required to deliver the interventions included within each intervention category. Expected costs were estimated separately for interventions delivered to groups versus those delivered to individuals as the latter are usually cheaper on a cost per patient basis. Information was extracted on the number of sessions, duration of sessions and the healthcare professionals involved in delivering or facilitating the interventions. For interventions delivered to groups, information was also extracted on the number of individuals who started the intervention, the group size and the number of groups (assumed to be one if not stated) to allow an estimation of the average cost per patient. Unit costs were taken from the Personal Social Services Research Unit (PSSRU) Unit Costs of Health and Social Care 2023^24^ with the exception of CBT, which was only reported in a previous edition of the PSSRU Unit Costs (2017),^25^ and therefore, this cost was uplifted to 2023 prices using the Hospital and Community Health Service Pay and Prices index based on PSSRU 2023. Unit costs used to estimate the expected intervention costs are summarised in the online supplemental table S2. Detailed assumptions used in the costing analysis on a study-by-study basis are provided in online supplemental tables S3–S8. Median costs across each intervention category were used in the economic analysis for the deterministic analysis. Interventions were typically shorter than 1 year, so discounting was not applied.

The costing analysis includes only intervention costs and does not estimate its impact on other healthcare resource use such as reduced primary or secondary care attendances resulting from patients experiencing lower fatigue or increased use from potential adverse effects. Studies identified in the cost-effectiveness review that assessed the overall impact of an intervention on healthcare costs have been reported elsewhere.^14^

### Cost-effectiveness

The cost-effectiveness analysis was not performed on a study-by-study basis. Instead, we focused on intervention categories that have been considered sufficiently similar to be analysed together within the NMA. The same effectiveness was applied across group and individual interventions in the base case as these were pooled within the NMA. A scenario analysis was conducted to test the validity of assuming equivalent treatment effects for group and individual interventions, using data for CBT-based fatigue interventions (CBT-Fatigue), where sufficient data were reported to estimate treatment effects separately (see online supplemental table S10 for detailed NMA results). The net monetary benefit (NMB) was estimated based on the willingness to pay threshold of £20 000 per QALY gained.^26^ The cost-effectiveness estimates were generated using both deterministic and probabilistic versions of the model. The deterministic model applied the point estimates of the parameters, whereas the probabilistic model used Monte Carlo sampling across 5000 iterations to generate distributions of expected health outcomes and costs for each treatment group. Uncertainty around treatment effect was handled using Convergence Diagnostics and Analysis (CODA) samples^27^ (see online supplemental appendix for further details) while baseline FSS score and regression coefficients were sampled from a normal distribution and the intervention costs from gamma distributions (see online supplemental table S11). When the variance-covariance matrices for the mapping coefficients were not reported in the paper, it was assumed that the coefficient for FSS was independent of the intercept parameter. As standard errors (SEs) of costs were unavailable, they were estimated using the lowest and highest expected costs of each intervention type across relevant studies. When the intervention cost was limited to a single study, the SE was assumed to be equal to 25% of the expected cost.

Scenario analyses were also conducted exploring the impact of (i) optimistic and pessimistic durations for treatment effect decline, including an assumption of immediate return of treatment effect to baseline after the last follow-up, (ii) alternative baseline FSS score and SD for FSS based on studies from the NMA EOT network, (iii) different SMDs (the effectiveness measure for fatigue) between individual and group CBT-Fatigue interventions, (iv) alternative SMDs using the shorter follow-up point in the LT network, (v) assuming that SMD of mindfulness at the ST follow-up time point is zero by removing the linear change assumption between EOT and LT, (vi) lower and upper costs and (vii) discount rates of 1.5% and 6%.

## Results

Based on the NMA results, physical activity promotion, CBT-Fatigue and mindfulness were found to be eligible for our economic analysis as they had RCTs reporting fatigue outcomes at >3 months after the EOT and they had a statistically significant SMD in fatigue scores versus usual care at either ST or LT follow-up. We chose not to include remote ischaemic conditioning in the economic analysis, as although this did achieve a statistically significant difference in fatigue versus usual care in the LT NMA, this was based on a single study in patients with stroke^28^ and the intervention was potentially specific to the population and so less likely to be generalisable to patients experiencing fatigue associated with other chronic conditions. It should be noted that the quantity of studies informing the NMA was greater for the CBT-Fatigue with 17 EOT, seven ST and nine LT studies informing the estimates. This compared with only seven EOT, one ST and two LT studies for physical activity promotion, and three EOT and one LT study for mindfulness. Although each intervention had been studied in more than one condition at EOT, only CBT-Fatigue provided evidence across a range of conditions at LT follow-up. Further details on the number of studies and the range of conditions for each intervention are provided in online supplemental tables S13 and S14.

### Health outcomes

#### Quality-adjusted life-years

**Figure 1:**
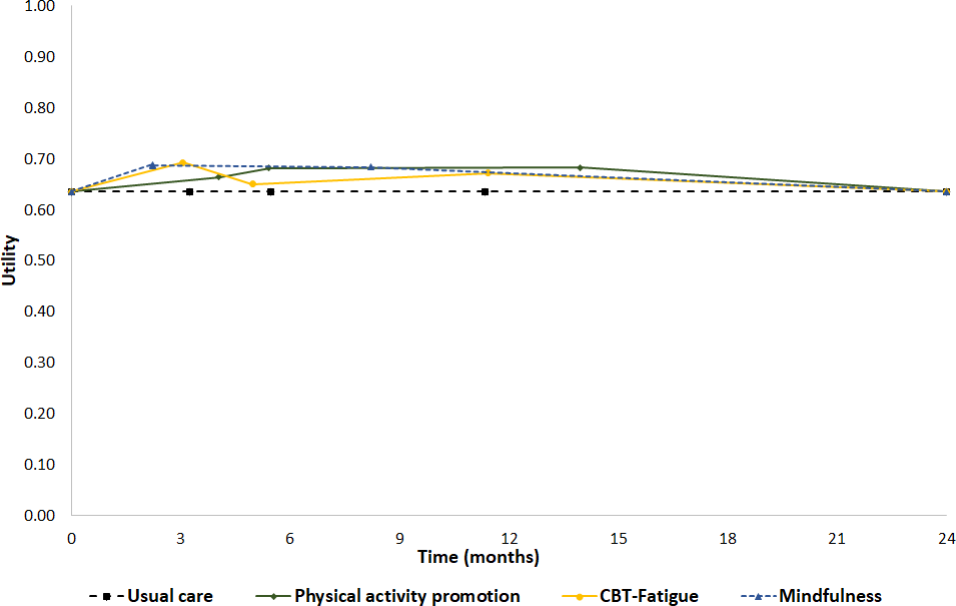
Utility values of interventions across different time points. CBT, cognitive–behavioural therapy.

The mapped SF-6D utility values assumed in the base-case analysis for interventions and the usual care are shown in figure 1 and those used in the scenario analyses are presented in online supplemental figures S1–S5. Physical activity promotion was estimated to have the highest utility values at ST and LT follow-up time points in both base-case and sensitivity analyses. Both probabilistic and deterministic base cases indicated that mindfulness and physical activity promotion interventions were associated with higher QALYs gained compared with CBT-Fatigue, with mindfulness slightly exceeding physical activity promotion (table 1).

**Table 1:** Base-case cost-effectiveness results

Intervention type	Group or individual intervention	Number of study arms	Median cost (£)	Mean QALYs (discounted)	Incremental costs	Incremental QALYs	NMB at £20 000 threshold	INMB against UC
Base-case (deterministic)								
UC	–	–	£0	1.227	–	–	£24 531	–
Physical activity promotion	Individual	3	£267	1.287	£267	0.060	£25 465	£934
CBT-Fatigue	Individual	10	£817	1.271	£817	0.045	£24 606	£75
Mindfulness	Individual	2	£465	1.287	£465	0.060	£25 272	£741
Physical activity promotion	Group	2	£156	1.287	£156	0.060	£25 576	£1045
CBT-Fatigue	Group	5	£484	1.271	£484	0.045	£24 939	£408
Mindfulness	Group	1	£215	1.287	£215	0.060	£25 522	£991
Base-case (probabilistic)								
UC	–	–	£0	1.223	–	–	£24 467	–
Physical activity promotion	Individual	3	£267	1.283	£267	0.060	£25 401	£934
CBT-Fatigue	Individual	10	£810	1.268	£810	0.045	£24 557	£90
Mindfulness	Individual	2	£462	1.284	£462	0.061	£25 226	£759
Physical activity promotion	Group	2	£157	1.283	£157	0.060	£25 512	£1045
CBT-Fatigue	Group	5	£485	1.268	£485	0.045	£24 882	£415
Mindfulness	Group	1	£214	1.284	£214	0.061	£25 474	£1007

CBT, cognitive–behavioural therapy; INMB, incremental net monetary benefit; NMB, net monetary benefit; QALY, quality-adjusted life years; UC, usual care.

#### Resource use and costs

**Figure 2:**
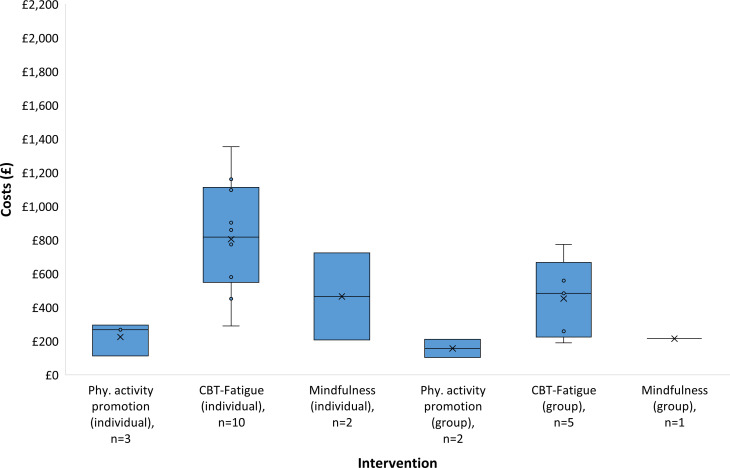
Distribution of costs by type of intervention. CBT, cognitive–behavioural therapy; Phy. activity, physical activity.

A cost was derived from at least one arm of 21 studies that included a total of 23 intervention arms that were classified as CBT-Fatigue, physical activity promotion or mindfulness. Costs could not be estimated for five studies (Nguyen *et al*,^29^ Kucharski *et al*,^30^ Okkersen *et al*,^31^ Pöttgen *et al*^32^ and Katz *et al*^33^) because no information was provided in the publications on the duration of sessions with healthcare providers or the interventions included only web-based tools for which no cost data were available. The range of costs for each intervention is presented in figure 2 as box-and-whisker plots. As summarised in table 1, the mean costs estimated by the probabilistic model were similar to the median costs from the deterministic model. The model suggests that group physical activity promotion incurred the lowest costs, whereas individual CBT-Fatigue had the highest costs due to higher staff contact time in terms of frequency and duration of sessions.

#### Cost-effectiveness

**Figure 3:**
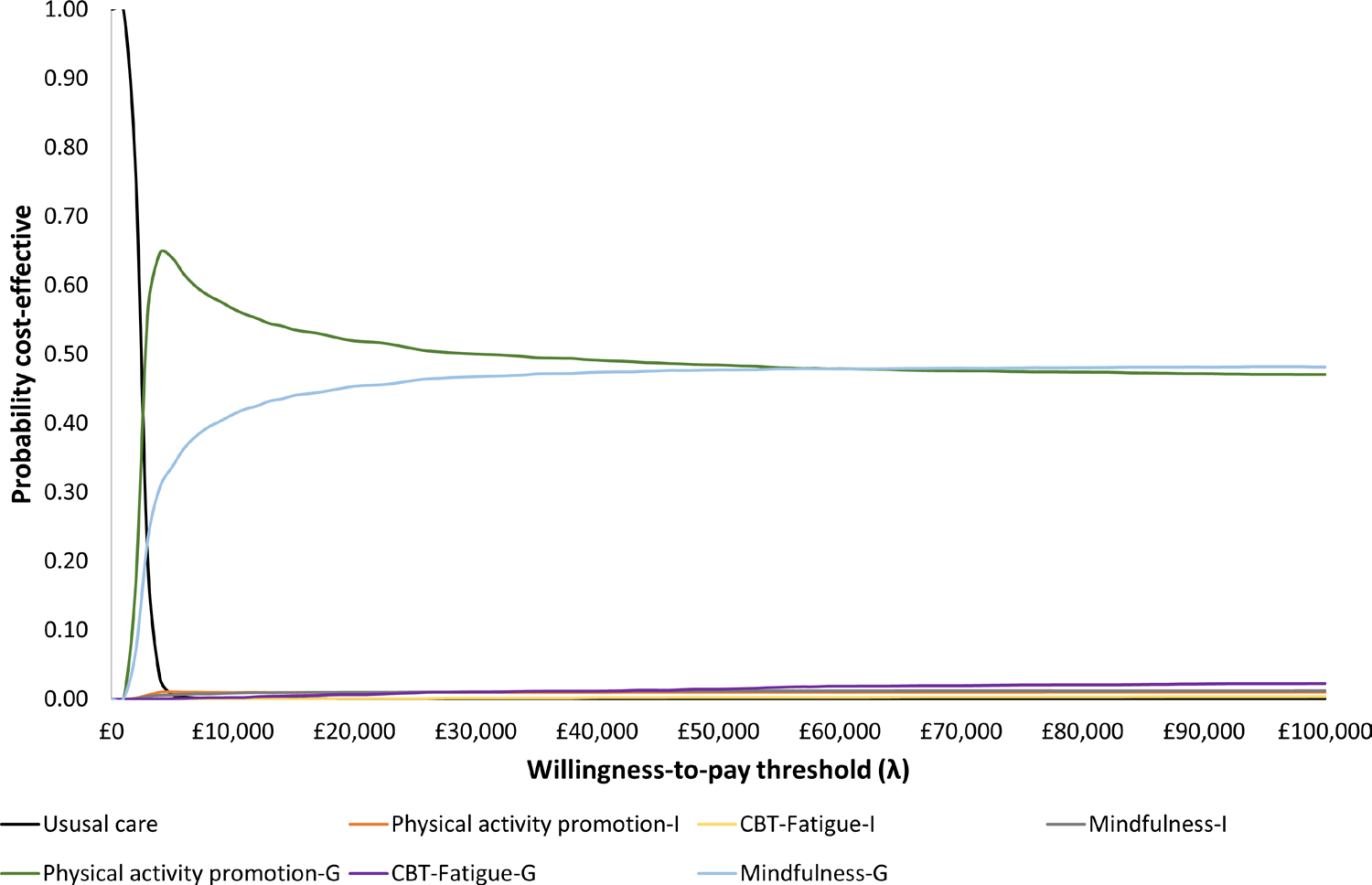
Multiway cost-effectiveness acceptability curves comparing non-pharmacological interventions and usual care. CBT, cognitive–behavioural therapy; CBT-Fatigue-G, CBT-Fatigue (group); CBT-Fatigue-I, CBT-Fatigue (individual); Mindfulness-I, mindfulness (individual); Mindfulness-G, mindfulness (group); Physical activity promotion-G, physical activity promotion (group); Physical activity promotion-I, physical activity promotion (individual).

The incremental costs and QALYs and the incremental net monetary benefit (INMB) for each intervention versus usual care, using deterministic and probabilistic models, are presented in table 1. It can be seen that for all interventions, the INMB versus usual care is positive, which means that the monetary value of the QALYs gained is greater than the intervention cost, indicating that the intervention would be considered cost-effective when valuing a QALY at £20 000. In both deterministic and probabilistic analyses, physical activity promotion (group intervention) is estimated to provide the highest INMB compared with usual care, followed by group mindfulness and then individual physical activity promotion. The higher cost of CBT-Fatigue interventions means that they have the lowest INMB versus usual care with individual CBT-Fatigue having the lowest INMB. The results of scenario analyses (online supplemental table S12) are similar to the base case for the majority of the interventions, with the exception being CBT-Fatigue (individual intervention). Due to the higher cost of CBT-Fatigue, it has a negative INMB, indicating an incremental cost-effectiveness ratio (ICER) >£20 000 per QALY versus usual care under certain conditions: (i) pessimistic assumption of the treatment effect decline, (ii) immediate return of treatment effect to the baseline after the last follow-up, (iii) alternative baseline FSS score and (iv) higher intervention costs. Cost-effectiveness planes representing pair-wise comparisons of each intervention versus usual care are presented in online supplemental figures S6–S11. At a willingness-to-pay threshold of £20 000 per QALY gained, at least 95% of probabilistic sensitivity analysis (PSA) simulations for all interventions, with the exception of CBT-Fatigue (individual), fall below the threshold line in the north-east quadrant of the cost-effectiveness planes, indicating that most interventions have at least a 95% probability of being cost-effective when compared with usual care. For CBT-Fatigue (individual), the probability of being cost-effective is 63%. A multiway cost-effectiveness acceptability curve (CEAC) was generated (figure 3) to assess the probability that each intervention is cost-effective compared with all competing interventions and usual care. The CEAC indicates that the probabilities of being cost-effective at a willingness-to-pay threshold of £20 000 per QALY gained are 52% for the physical activity promotion (group intervention) and 45% for the mindfulness (group intervention), while the other interventions have probabilities below 1%.

## Discussion

A recent systematic literature review by Davis *et al*^16^ identified that the cost-effectiveness evidence available for non-pharmacological interventions for fatigue in patients with LT conditions is limited to a small number of within-trial analyses.^2234^,^36^ These analyses examined a limited number of interventions (CBT-Fatigue and physical activity promotion) in a narrow range of populations (multiple sclerosis and inflammatory rheumatic conditions). The economic literature is therefore not reflective of the broader literature on clinical effectiveness, which covers a greater range of conditions and interventions.^14^ To our knowledge, no existing study has used decision analytic modelling to assess the relative cost-effectiveness of non-pharmacological interventions by drawing on this broader clinical effectiveness literature.^14^ Our de novo economic assessment evaluated the cost-effectiveness of interventions that had been found, in a systematic review and NMA of interventions across LT medical conditions, to demonstrate a statistically significant improvement in fatigue beyond the EOT. The base-case model suggests that physical activity promotion, CBT-Fatigue and mindfulness could all be cost-effective when compared with usual care at the willingness-to-pay threshold of £20 000 per QALY gained, regardless of whether they are delivered individually or in groups. However, it should be noted that the analysis pools effectiveness data across group and individual interventions but estimates costs separately. These findings were robust under the scenario analyses conducted with the exception of CBT-Fatigue delivered to individuals, which had the lowest INMB in the base case and was therefore less robust to scenarios with more pessimistic assumptions.

### Strengths and limitations

One of the key strengths of our analysis is that it was based on the pooled results of fatigue outcomes across multiple LT conditions, derived from a comprehensive systematic review and NMA. Studies on multiple sclerosis accounted for around half of the included studies in each network. Both base-case and scenario analyses are probabilistic, capturing the model parameter uncertainty. In addition, the probabilistic model explicitly incorporated the correlation between efficacy estimates (fatigue outcomes) using the CODA samples generated by the NMA.

Our economic assessment is also subject to some limitations. Instead of exploring the clinical effectiveness and cost-effectiveness of each intervention on a study-by-study basis, the evidence was synthesised at the intervention category level by pooling the treatment effects across studies assessing similar interventions. This approach may produce aggregation bias as important between-study differences may be masked. However, we considered that summarising interventions into coherent categories was valuable for decision-making, as it allowed us to integrate all available evidence into a form usable for the cost-effectiveness model, as opposed to using study-specific estimates. To mitigate heterogeneity, a random-effects NMA was implemented,^14^ which explicitly models between-study variability and therefore helps to accommodate differences in populations, delivery and study design. Within the PSA, the potential heterogeneity in the treatment effect estimate was explored further.

Very few studies reported the UK estimates of resource use other than those associated with delivering the intervention and therefore, the estimated incremental costs might be overestimated or underestimated if the interventions result in decrease or increase in other resource use. Additionally, a detailed cost breakdown for internet-based or app-based tools (eg, MS Invigor8,^34^ ELEVIDA^32^ and Tailorbuilder)^37^ was unavailable; therefore, the cost of such tools was excluded from the model, which might lead to an underestimation of costs for some interventions, particularly CBT-Fatigue and mindfulness. In terms of intervention costs per patient, interventions delivered to groups were found to be less costly than the same type of interventions delivered individually. For group interventions, group size determined the cost per patient. Where the group size was not reported in the studies, it was assumed that there was a single group and that may have underestimated the cost per patient if smaller groups were used instead.

There is a broad range of costs for each type of intervention across included studies, indicating a high level of heterogeneity in expected cost estimates. Sensitivity analyses explored the impact of the heterogeneity in intervention costs, and only CBT-Fatigue (individual) was found to have an ICER >£20 000 per QALY versus usual care when the highest cost was applied. This finding aligns with the results from three within-trial economic evaluations identified in a recent systematic review. Thomas *et al*,^35^ Chong *et al*^36^ and Hewlett *et al*^22^ found that CBT was dominated by usual care because of higher intervention costs and small negative incremental QALYs. Chong *et al*^36^ compared physical activity promotion to CBT directly using a within-trial analysis of the RCT (reported by Bachmair *et al*^38^) and their conclusion that CBT was dominated by physical activity promotion is replicated in our de novo analysis informed by effectiveness data from multiple studies. Given that CBT-Fatigue may include elements of physical activity promotion or mindfulness, these findings raise the question of whether there is ‘added value’ from the additional cognitive components of CBT-Fatigue interventions, given that these are generally more intensive to deliver. The existing study by Chong *et al* does not fully answer this question because it did not include a mindfulness-based intervention. In addition, its generalisability to patients with a broad range of chronic conditions may be limited as it only included patients with inflammatory rheumatic diseases.

The expected QALYs were based on the assumption that the difference between interventions and usual care would start to decline after the last follow-up time point and become zero at 24 months (from the baseline). This assumption was tested using optimistic and pessimistic scenarios regarding the treatment effect dissipation. All interventions were less cost-effective when making a more pessimistic assumption about the persistence of the treatment effect but only CBT-Fatigue (individual) had an ICER >£20 000 per QALY when assuming that the treatment effect declined rapidly within a year after the intervention. Another limitation is that the probabilistic model had to use SEs for mapping coefficients when sampling from a normal distribution instead of using variance-covariance matrix, which might overestimate the uncertainty of the mapping algorithm. In addition, the mapping algorithm reported by Goodwin *et al* was derived in a multiple sclerosis population, introducing uncertainty regarding the generalisability of SF-6D estimates for the broader modelled population of patients with LT conditions.

The scenario analysis exploring the impact of allowing for different treatment effects for CBT-Fatigue from group and individual interventions had minimal impact on the cost-effectiveness estimate for CBT-Fatigue versus usual care due to prediction of similar treatment effects. However, it should be noted that we were unable to conduct a similar analysis exploring the impact of allowing for different treatment effects of group and individual interventions for the mindfulness and physical activity promotion. This was due to there only being data beyond the EOT available for group mindfulness interventions and individual physical activity promotion interventions. As such, the cost-effectiveness results for individual mindfulness interventions and group physical activity promotion interventions should be interpreted with caution.

Although our analysis is able to estimate which interventions have the highest INMB, any head-to-head comparison should be interpreted with caution given the heterogeneity in intervention costs across the included studies as shown by the overlapping CIs for the intervention costs. However, across all the scenario analyses explored, CBT-Fatigue had lower QALY gains than mindfulness or physical activity promotion due to a smaller and non-statistically significant difference in fatigue scores at the ST follow-up estimated in the NMA. It should also be noted that no efficacy data were available for mindfulness from the ST follow-up and the assumption of a linear change in fatigue between the EOT and the LT follow-up point for the mindfulness intervention could be optimistic. However, in our scenario analysis we found that mindfulness had greater QALY gains than CBT-Fatigue even when assuming no clinical efficacy at the ST follow-up point for mindfulness. The analysis for CBT-Fatigue was also the only analysis in which there were data from multiple studies at both the ST and LT follow-up points and in which the studies informing the LT follow-up were conducted across more than one type of chronic condition (five studies for multiple sclerosis, one for stroke, two for musculoskeletal disease and one for inflammatory bowel disease). Therefore, the greater QALY gains for mindfulness and physical activity promotion in this analysis should not be overinterpreted as demonstrating clinical superiority for patients with chronic conditions, as the LT data for these interventions are based on a smaller set of studies covering a limited set of conditions (two studies for physical activity promotion in musculoskeletal conditions and one study for mindfulness in multiple sclerosis). Additionally, Leaviss *et al*^14^ concluded that the strength of clinical effectiveness evidence is moderate for physical activity promotion and low for CBT-Fatigue and mindfulness due to heterogeneity between studies and high risk of bias. These Grading of Recommendations, Assessment, Development and Evaluations (GRADE) ratings reflect the strict application of the Cochrane Risk of Bias V.2.0 tool,^39^ with evidence generally judged to be at high risk of bias due to methodological issues inherent in evaluating behavioural interventions such as impossibility of blinding.

## Conclusions

This de novo economic evaluation indicates that mindfulness interventions, physical activity promotion interventions and CBT-Fatigue interventions have the potential to be a cost-effective means for improving quality of life in people experiencing fatigue associated with a chronic condition when compared with usual care. If it is assumed that group and individual interventions have similar efficacy, as supported by our analysis of CBT-Fatigue interventions, then group interventions tend to be lower cost to deliver and are therefore more cost-effective than individual interventions. However, there is still uncertainty regarding the differences in treatment effects between individual and group interventions for physical activity promotion and mindfulness due to the lack of studies reporting outcomes beyond the EOT for individual mindfulness interventions and group physical activity promotion interventions. While CBT-Fatigue had higher costs and lower QALY gains than the other interventions, the clinical effectiveness estimates for the other interventions are based on fewer studies conducted across a narrower range of conditions. Therefore, while our analysis suggests that CBT-Fatigue has a low probability of being most cost-effective when comparing the different interventions against each other, we consider that there remains some uncertainty as to which non-pharmacological fatigue management intervention would be most cost-effective when used across a population of patients with a diverse range of LT conditions. In addition, the strength of clinical effectiveness evidence was graded as moderate for physical activity promotion and low for CBT-Fatigue and mindfulness. We therefore recommend that future research is conducted to provide a head-to-head comparison of the cost-effectiveness of CBT-Fatigue, physical activity promotion and mindfulness interventions across a broad population with different LT conditions.

## Supplementary material

10.1136/bmjopen-2025-102846 online supplemental file 1

## Data Availability

All data relevant to the study are included in the article or uploaded as supplementary information.
