# Supplementary material for: Model-based economic evaluation of non-pharmacological interventions for fatigue in patients with long-term medical conditions in the UK
Source: BMJ Open. 2026 Apr 24;16(4):e102846. doi: 10.1136/bmjopen-2025-102846 (PMC13110622; doi:10.1136/bmjopen-2025-102846)
Supplement: Supplementary file 2 [file bmjopen-2025-102846.reviewer_comments.pdf]

PEER REVIEW HISTORY

BMJ Open publishes all reviews undertaken for accepted manuscripts. Reviewers are asked to complete a checklist review form and are provided with free text boxes to elaborate on their assessment. These free text comments are reproduced below.

ARTICLE DETAILS

Title (Provisional)

A model-based economic evaluation of non-pharmacological interventions for fatigue in patients with long-term medical conditions in the United Kingdom

Authors

Mon-Yee, Mon; Burton, Christopher D; Leaviss, Joanna; Forsyth, Jessica Eve; Daly, George; Davis, Sarah

---

VERSION 1 - REVIEW

**Reviewer**	**1**
**Name**	**Nystrand, Camilla**
**Affiliation**	**Karolinska Institute, Department of Learning, Informatics, Management and Ethics**
**Date**	**09-May-2025**
**COI**	**None**

Thank you for the opportunity to review this manuscript, which is aiming to assess the cost-effectiveness of different types of interventions to treat fatigue. The authors have used a systematic approach to identify and select relevant studies that have assessed intervention effectiveness, grouped and analyzed these studies by type of intervention (CBT, mindfulness, physical activity), assessed the costs of the interventions and conducted CEAs of these three types of interventions. To improve the manuscript and allow the reader to fully understand the paper, the following is suggested:

The introduction is rather short, and it leaves the reader with quite a few questions. The authors should consider extending this section, and at least include the following:

• information about which patient groups that experience fatigue

• how prevalent it is

• something about the disease panorama/how it progresses

• the short- and long-term effects of interventions

• the healthcare/societal economic burden of fatigue.

Page 4, row 9-10: “The experience of fatigue appears to show similarities across different conditions” – which similarities?

It is also unclear to the reader why this study is important, given what evidence is already available.

One of the aims of the study is to “identify the interventions that would warrant further investigation in future cost-effectiveness studies”. It is unclear how the authors have attempted to answer this aim, and on what grounds these interventions would be identified. Also, the authors make a conclusion that “CBT-Fatigue, physical activity promotion and mindfulness interventions all demonstrated the potential to be cost-effective versus usual care. Future research is recommended to compare the cost-effectiveness of these interventions across a broad population with different chronic conditions.” However, this conclusion does not seem to be supported by any results found in this study. A suggestion is thus to either remove this aim, or to explicitly add to the methods, results and discussion how this aim has been addressed and what has been found.

:

The authors have assumed that a waning of the treatment effects post the last follow-up. It is suggested to add a sensitivity analysis for a treatment effect of zero after the last follow-up, as a it would mirror the data availability.

It is unclear why the authors have chosen the specific time horizons used in the study.

Please add a reference to the use of £20,000 as the willingness-to-pay threshold?

It is unclear whether the authors used the FSS as the only relevant outcome from the clinical effectiveness papers, that was then mapped to SF-6D. If so, what was the reason?

Unit costs for CBT: Were the unit costs unrelated to the type of healthcare professional and whether it is provided as face-to-face or distance? Are cost of facilities included in the unit cost of CBT? Was indirect time accounted form?

In Table S4, both singular and plural forms of the healthcare professionals are used. Is this on purpose, and have the costs been estimated based on this? If yes, please add the quantity of healthcare staff needed to perform the intervention.

Table S6 – why is only staff time included in the costing, and not other cost components, such as a facility cost?

Are there any other resources needed to deliver the interventions in general? Materials, IT, training of staff, overhead, etc.

It is suggested to vary the discount rate in a scenario analysis, as this is often proposed in guidelines (and in CHEERS).

It would be of value if the authors could report the utility estimates at each time-point in the S11 table.

It is unclear which articles that the final effect estimate for each intervention was based on. Is it all interventions mentioned in the costing tables? It would help the reader if these interventions were listed in a table, along with relevant information, such as target population, age groups, setting, country, etc.

The authors state that they removed one intervention, as it was deemed not generalizable. If generalizability of the intervention was a criterion for inclusion, it is relevant to include a description of what is meant by generalizability and for each study, how it has been assessed as generalizable. Also, the excluded study should be cited.

It is suggested that the probabilistic basecase results are shown in a cost-effectiveness plane, as well as on a CEAC.

Page 11, row 7-8: "To our knowledge, no study has assessed the cost effectiveness of such interventions across multiple conditions using decision analytic modelling". It is unclear to the reader what condition have been assessed in this study, and why this would be of relevance/importance.

Page 11, row 13-18: "The base-case model suggests that physical activity promotion, CBT-Fatigue and mindfulness could all be cost-effective at the willingness-to-pay threshold of £20,000 per QALY gained, regardless of whether they are delivered individually or in groups." I believe this is a strong argument, as the effects have not been assessed based on these groupings (only for CBT in a scenario analysis), only the costs. The authors also state that one should interpret the results carefully for individual mindfulness intervention and group physical activity promotion later in the discussion…

Page 13, row 3-9: "The analysis for CBT-Fatigue was also the only analysis in which there was data from multiple studies at both the ST and LT follow-up points and in which the studies informing these endpoints were conducted across more than one type of chronic condition (nine studies for multiple sclerosis, three for stroke, three for musculoskeletal disease and one for inflammatory bowel disease)". This information regarding the different types of chronic conditions is new to the reader, and ought to have been brought up earlier to warrant a discussion about it.

There is some comparison to economic evaluations of CBT for fatigue, but little other comparisons to results from other economic evaluations. The manuscript would benefit from extending this discussion, also in light of the missing discussion on the implications of these results for policy and practice, as well as future research.

The use of mapping algorithms for the estimation of utilities and QALYs comes with limitations and should be brought up for discussion.

Page 13, row 37-40: "We therefore recommend that future research is conducted to compare the cost-effectiveness of CBT Fatigue, physical activity promotion and mindfulness interventions across a broad population with different chronic conditions." It is unclear, from

the discussion of the paper, what the authors mean by this conclusion and why they conclude with this.

Reviewer **2**

Name **Muflih, Suhaib**

Affiliation **Jordan University of Science and Technology, Clinical Pharmacy**

Date **13-Nov-2025**

COI **None**

Very interesting study that highlights the need for conducting cost-effectiveness analysis when implementing non-pharmacological approaches. I have a few minor comments that I believe will further enhance the quality of the paper:

• Please note that some abbreviations need to be spelled out on first mention, such as CBT-Fatigue, CIS-F, HERC database…

• Could you clarify how you could exclude the existence of pharmacological therapy in the intervention arms to minimize selection bias.

• The application of existing algorithms derived from MS population might produce inaccuracies at the bottom of the SF-6D scale, due to the different severity between MS and other chronic diseases. Should it be addressed in the limitation?

• Can you provide statistical performance indicators (e.g., R2 or RMSE) to support the following sentence: "The regression model mapping from total FSS to SF-6D was reported as performing better than the regression model mapping to EQ-5D."

• The decision to conduct the analysis on intervention categories rather than on a study-by-study basis is a limitation, potentially leading to aggregation bias. By pooling interventions deemed similar, the NMA may have concealed substantial heterogeneity across studies (e.g., in patient characteristics or intervention logistics), thus compromising the internal validity and generalizability of the conclusions. Please discuss in the limitation section.

• The use of Convergence Diagnostics and Analysis (CODA) samples is noted, but the manuscript lacks an adequate description of the specific convergence assessment procedures employed and the detailed process by which posterior samples were integrated into the economic model. Please provide more details.

VERSION 1 - AUTHOR RESPONSE

**Reviewer's comments**	**Authors' responses**
**Reviewer 1**	
Introduction	

The introduction is rather short, and it leaves the reader with quite a few questions. The authors should consider extending this section and at least include the following:<br>• information about which patient groups that experience fatigue<br>• how prevalent it is<br>• something about the disease panorama/how it progresses<br>• the short- and long-term effects of interventions<br>• the healthcare/societal economic burden of fatigue.	We have added text to the introduction discussing the prevalence and burden of fatigue in patients with long-term conditions and a sentence summarising the findings on long-term treatment effectiveness.
Page 4, row 9-10: "The experience of fatigue appears to show similarities across different conditions" – which similarities?<br>It is also unclear to the reader why this study is important, given what evidence is already available.	We acknowledge the reviewer's suggestions and have expanded the text in the introduction section.
• One of the aims of the study is to "identify the interventions that would warrant further investigation in future cost-effectiveness studies".<br>• It is unclear how the authors have attempted to answer this aim, and on what grounds these interventions would be identified.<br>• Also, the authors make a conclusion that "CBT-Fatigue, physical activity promotion and mindfulness interventions all demonstrated the potential to be cost-effective versus usual care.<br>• Future research is recommended to compare the cost-effectiveness of these interventions across a broad population with different chronic conditions."<br>• However, this conclusion does not seem to be supported by any results found in this study.<br>• A suggestion is thus to either remove this aim, or to explicitly add to the methods, results and discussion how this aim has been addressed and what has been found.	We've removed this as an explicit aim as suggested.
Methods	
The authors have assumed that a waning of the treatment effects post the last follow-up. It is suggested to add a sensitivity analysis for a treatment effect of zero after the last follow-up, as it would mirror the data availability.	We have explored the scenario as suggested. The results are presented in Table S13 and Figure S3, and the findings are discussed in the main paper.
It is unclear why the authors have chosen the specific time horizons used in the study.	A 24-month time horizon in the base case was informed by findings from studies (Gay et al.[1] and Hewlett et al.[2]), which suggest that there is a potential for treatment effects to remain beyond one year after stopping the treatment. As durations of most interventions evaluated in our model were less than 12 months, a time horizon of 24 months was chosen. To test this

	uncertainty, optimistic and pessimistic scenarios were conducted. The range tested was informed by clinical expert advice having been presented with the findings reported by Gay et al. at 18 months and Hewlett et al. at 24 months.<br><br>We have added a statement to the methods section (quality-adjusted life-years) to clarify the rationale for choosing these time horizons.
Please add a reference to the use of £20,000 as the willingness-to-pay threshold?	A reference (NICE health technology evaluations: the manual) has been added as suggested.
It is unclear whether the authors used the FSS as the only relevant outcome from the clinical effectiveness papers, that was then mapped to SF-6D. If so, what was the reason?	Among the various fatigue scales used in the studies included in the NMA, the FSS was selected based on clinical input and the availability of the mapping algorithm for converting fatigue outcomes into health state utility values.<br><br>We have edited the text describing the selection of the mapping algorithm to make it clearer that we didn't have an a priori preference for mapping algorithms that used FSS, but that the other available mapping algorithms which included other fatigue measures were less suitable.<br><br>We have also amended the methods section to better explain that the NMA pooled data from studies irrespective of the fatigue tool used and the treatment effects were converted to differences in FSS due to the availability of the mapping algorithm.
Unit costs for CBT: Were the unit costs unrelated to the type of healthcare professional and whether it is provided as face-to-face or distance? Are cost of facilities included in the unit cost of CBT? Was indirect time accounted for?	The unit cost of CBT was taken from PSSRU 2017, which estimated the unit costs by taking account of staff salaries (weighted wages for a speciality doctor, a clinical psychologist and a mental health nurse), overheads, capital costs, working time, the ratio of direct to indirect time on face-to-face contact and length of contact. Therefore, we assumed that the reported unit costs were comprehensive and did not need additional adjustments to account for the type of professional and indirect staff time.<br>We have added additional footnotes to Table S2 to describe the detailed calculations of unit costs taken from PSSRU.

In Table S4, both singular and plural forms of the healthcare professionals are used. Is this on purpose, and have the costs been estimated based on this? If yes, please add the quantity of healthcare staff needed to perform the intervention.	Table S4 only summarises types of healthcare professionals used in those studies. For the calculations of expected costs, the unit cost of CBT was used across all studies which provides the cost per staff member per minute of face-to-face activity. If more than one staff member was required this was accounted for as shown in Table S7 where it notes 2 staff members for Gay et al., 2 staff members from Hewlett et al. and 2 staff members for Thomas et al.[3] and in Table S6 where it notes 2 staff members per group for Lutz et al.[4] However, none of the interventions in Table S4 required 2 staff members. We've also corrected the expected costs for CBT in Bredero et al.[5] where 2 trainers had been costed previously. This correction has a minimal impact on the intervention's cost-effectiveness results with the INMB vs UC changing by only £1 in the base case analysis (absolute NMBs at £20,000 vary slightly from previous results due to a correction in the random number control for one parameter).<br><br>We have added a footnote to Table S4 to clarify that the unit cost of CBT was used, as it reflects a weighted average three different health professionals and accounts for the direct to indirect time ratio. We also added a footnote to Tables S3 to S8 and quantified the number of staff in the unit cost column where relevant.
• Table S6 – why is only staff time included in the costing, and not other cost components, such as a facility cost?<br><br>• Are there any other resources needed to deliver the interventions in general? Materials, IT, training of staff, overhead, etc.	The overhead costs were already included in the estimation of unit costs of a physiotherapist.<br><br>We have added a footnote to Table S2 explaining the calculation methods used in PSSRU, as described in the above response.
It is suggested to vary the discount rate in a scenario analysis, as this is often proposed in guidelines (and in CHEERS).	We have explored two additional scenarios using 1.5% and 6% for health outcomes, and the results are presented in Table S12.
It would be of value if the authors could report the utility estimates at each time-point in the S11 table.	Table S11 reports inputs to the analysis, and the utility values at each time point are not inputs as they are derived by applying the mapping algorithm to the fatigue outcomes already reported in Table S11. The utility values generated within the model are already presented for reference in Figure 1 of the main paper and Figures S1 to S5 of the supplementary materials.
It is unclear which articles that the final effect estimate for each intervention was based on. Is it all interventions mentioned in the costing tables?	In this modelling study, only interventions that were found to be statistically significant in the NMA were evaluated, and therefore only

It would help the reader if these interventions were listed in a table, along with relevant information, such as target population, age groups, setting, country, etc.	studies relevant to these interventions were mentioned in the costing tables. However, the treatment effect estimates for these interventions were informed by the full NMA network presented in Leaviss et al.,[6] including those which were not statistically significant. All interventions and studies included in the NMA for are presented in a separate paper, and we have now added a preprint version of it to the reference list. We also have added a statement referring the reader to the NMA for further details on the included studies. However, as the reviewer appears to want more information on the studies available for the three interventions modelled, we have summarised these in Tables S13 to S14.
Results	
The authors state that they removed one intervention, as it was deemed not generalizable. If generalizability of the intervention was a criterion for inclusion, it is relevant to include a description of what is meant by generalizability and for each study, how it has been assessed as generalizable. Also, the excluded study should be cited.	We acknowledge the reviewer' comments and have added a statement to the methods section to clarify that studies which were considered based on clinical advice not to be generalisable across different chronic conditions were excluded. The excluded study has also been cited in the results section.
It is suggested that the probabilistic base case results are shown in a cost-effectiveness plane, as well as on a CEAC.	Cost-effectiveness (CE) planes and CEAC have been added as suggested. (Figures S6 to S11 for CE planes and Figure 3 for CEAC). There are six CE planes, and each represents a pair-wise comparison of an intervention versus usual care.
Discussion	
Page 11, row 7-8: "To our knowledge, no study has assessed the cost effectiveness of such interventions across multiple conditions using decision analytic modelling". It is unclear to the reader what condition have been assessed in this study, and why this would be of relevance/importance.	We have amended the text in the discussion and added a recent systematic literature review of cost-effectiveness studies of non-pharmacological interventions across chronic conditions by Davis et al.[7] to the reference list, to further support the relevance of this modelling study.
Page 11, row 13-18: "The base-case model suggests that physical activity promotion, CBT-Fatigue and mindfulness could all be cost-effective at the willingness-to-pay threshold of £20,000 per QALY gained, regardless of whether they are delivered individually or in groups." I believe this is a strong argument, as the effects have not been assessed based on these groupings (only for CBT in a scenario analysis), only the costs. The authors also state that one should interpret the results carefully for individual mindfulness intervention and group physical activity promotion later in the discussion…	This sentence has been modified to make it clearer that the analysis relies on effectiveness data pooled across group and individual interventions.

Page 13, row 3-9: “The analysis for CBT-Fatigue was also the only analysis in which there was data from multiple studies at both the ST and LT follow-up points and in which the studies informing these endpoints were conducted across more than one type of chronic condition (nine studies for multiple sclerosis, three for stroke, three for musculoskeletal disease and one for inflammatory bowel disease)”. This information regarding the different types of chronic conditions is new to the reader, and ought to have been brought up earlier to warrant a discussion about it.	We acknowledge the reviewer’s comments and have added a definition of long-term medical conditions in the Methods section to make that we are considering a diverse range of conditions. This definition is consistent with the inclusion and exclusion criteria used in the clinical and cost-effectiveness review studies.<br><br>We have also added a sentence to the results to further describe the quantity of evidence contributing to the NMA for each intervention. Additionally, we added Tables S13 and S14 summarising studies for each intervention included in the economic model at EOT, ST and LT follow-up timepoints.
There is some comparison to economic evaluations of CBT for fatigue, but little other comparisons to results from other economic evaluations. The manuscript would benefit from extending this discussion, also in light of the missing discussion on the implications of these results for policy and practice, as well as future research.	The second paragraph on page 14 discusses all of the existing cost-effectiveness evidence identified in the published review including the study by Chong et al.[8] which compared CBT-Fatigue directly to physical activity promotion. The paragraph has been amended to make it clearer that the studies mentioned were identified in a systematic review and additional information has been added to the start of the discussion section to better describe the limits of the existing evidence base identified in the review. A more detailed discussion of the limitations of the study by Chong et al. and why further research is still needed has also been added.
The use of mapping algorithms for the estimation of utilities and QALYs comes with limitations and should be brought up for discussion.	We acknowledge the reviewer’s comments and have added a discussion around the usage of mapping algorithms to the discussion section.
Conclusion	
Page 13, row 37-40: “We therefore recommend that future research is conducted to compare the cost-effectiveness of CBT Fatigue, physical activity promotion and mindfulness interventions across a broad population with different chronic conditions.” It is unclear, from the discussion of the paper, what the authors mean by this conclusion and why they conclude with this.	We have added some additional text to make it clearer that whilst our analysis suggests that each of the three interventions has the potential to be cost-effective, we consider that there remains some uncertainty as to which non-pharmacological fatigue management intervention would be most cost-effective when used across a population of patients with a diverse range of long-term conditions. We believe our findings and their limitations support the need for a head-to-head comparison of these interventions across a population with a range of long-term conditions and have amended this last sentence to make this clearer.

**Reviewer 2**	
Please note that some abbreviations need to be spelled out on first mention, such as CBT-Fatigue, CIS-F, HERC database…	These abbreviations have been updated accordingly.
Could you clarify how you could exclude the existence of pharmacological therapy in the intervention arms to minimize selection bias.	Our economic model was part of, and mainly informed by, an evidence synthesis examining both the clinical and cost-effectiveness of non-pharmacological interventions for fatigue in long-term medical conditions. The effectiveness and cost-effectiveness of pharmacological interventions for fatigue were therefore outside of the scope of the review and the systematic review and NMA that informed the cost-effectiveness analysis excluded studies that solely compared drug interventions with non-drug interventions for fatigue. Studies that included pharmacological therapy for the underlying health condition were not excluded. The purpose of the review was to assess the effectiveness of non-pharmacological interventions to manage fatigue in patients receiving standard care treatment for their underlying condition, which would be expected to be similar between study arms, thus minimising any bias.<br>We have added a statement to make this explicit in the first paragraph of the methods section.
The application of existing algorithms derived from MS population might produce inaccuracies at the bottom of the SF-6D scale, due to the different severity between MS and other chronic diseases. Should it be addressed in the limitation?	We acknowledge the reviewer's comments and have added a statement to the limitation section to make it clear that the mapping algorithm reported by Goodwin et al.[9] was derived from the MS population, introducing uncertainty regarding the generalisability of SF-6D estimates for the modelled population.
Can you provide statistical performance indicators (e.g., R2 or RMSE) to support the following sentence: "The regression model mapping from total FSS to SF-6D was reported as performing better than the regression model mapping to EQ-5D."	These values have been added as suggested.
The decision to conduct the analysis on intervention categories rather than on a study-by-study basis is a limitation, potentially leading to aggregation bias. By pooling interventions deemed similar, the NMA may have concealed substantial heterogeneity across studies (e.g., in patient characteristics or intervention logistics), thus compromising the internal validity and generalizability of the conclusions. Please discuss in the limitation section.	We agree that synthesising evidence at the intervention-category level can introduce the possibility of aggregation bias, as important between-study differences may be masked. However, we considered that summarising interventions into coherent categories was valuable for decision-making, as it allowed us to integrate all available evidence into a form usable for the cost-effectiveness model, as opposed to using study specific estimates. To mitigate heterogeneity, we implemented a random-effects NMA, which explicitly models

	between-study variability and therefore helps to accommodate differences in populations, delivery, and study design. Within the PSA the potential heterogeneity in the treatment effect estimate is explored further.<br><br>This has however been noted as a potential limitation in the discussion.
The use of Convergence Diagnostics and Analysis (CODA) samples is noted, but the manuscript lacks an adequate description of the specific convergence assessment procedures employed and the detailed process by which posterior samples were integrated into the economic model. Please provide more details.	We acknowledge the reviewer's suggestions and have added a separate section to the supplementary appendix, explaining the detailed process for generating CODA samples.

1. Gay MC, Cassedanne F, Barbot F, et al. Long-term effectiveness of a cognitive behavioural therapy (CBT) in the management of fatigue in patients with relapsing remitting multiple sclerosis (RRMS): a multicentre, randomised, open-label, controlled trial versus standard care. *J Neurol Neurosurg Psychiatry* 2024;95(2):158-66. doi: 10.1136/jnnp-2023-331537 [published Online First: 20240111]
2. Hewlett S, Almeida C, Ambler N, et al. Group cognitive-behavioural programme to reduce the impact of rheumatoid arthritis fatigue: the RAFT RCT with economic and qualitative evaluations. *Health Technol Assess* 2019;23(57):1-130. doi: 10.3310/hta23570
3. Thomas S, Thomas PW, Kersten P, et al. A pragmatic parallel arm multi-centre randomised controlled trial to assess the effectiveness and cost-effectiveness of a group-based fatigue management programme (FACETS) for people with multiple sclerosis. *J Neurol Neurosurg Psychiatry* 2013;84(10):1092-9. doi: 10.1136/jnnp-2012-303816 [published Online First: 20130521]
4. Lutz C, Kersten S, Haas CT. Short-Term and Long-Term Effects of an Exercise-Based Patient Education Programme in People with Multiple Sclerosis: A Pilot Study. *Multiple Sclerosis International* 2017;2017(1):2826532. doi: https://doi.org/10.1155/2017/2826532
5. Bredero QM, Fleer J, Smink A, et al. Mindfulness-Based Cognitive Therapy for Fatigue in Patients with Inflammatory Bowel Disease: Results of a Randomized Controlled Trial. *Mindfulness* 2023;14(1):19-32. doi: 10.1007/s12671-022-02057-5
6. Leaviss J, Booth A, Coyle D, et al. Effectiveness of non-pharmacological Interventions For Fatigue in Long term conditions (EIFFEL)-systematic review and network meta-analysis. *medRxiv* 2025:2025.07.23.25332061. doi: 10.1101/2025.07.23.25332061
7. Davis S, Mon-Yee M, Sutton A, et al. Cost effectiveness of non-pharmacological interventions for fatigue in patients with long-term conditions: a systematic literature review. *Expert Review of Pharmacoeconomics & Outcomes Research* 2025;25(8):1159-66. doi: 10.1080/14737167.2025.2537194
8. Chong HY, McNamee P, Bachmair EM, et al. Cost-effectiveness of cognitive behavioural and personalized exercise interventions for reducing fatigue in inflammatory rheumatic diseases. *Rheumatology (Oxford)* 2023;62(12):3819-27. doi: 10.1093/rheumatology/kead157
9. Goodwin E, Hawton A, Green C. Using the Fatigue Severity Scale to inform healthcare decision-making in multiple sclerosis: mapping to three quality-adjusted life-year

measures (EQ-5D-3L, SF-6D, MSIS-8D). *Health and Quality of Life Outcomes* 2019;17(1):136. doi: 10.1186/s12955-019-1205-y

---

VERSION 2 - REVIEW

Reviewer **1**
Name **Nystrand, Camilla**
Affiliation **Karolinska Institute, Department of Learning, Informatics, Management and Ethics**
Date **05-Jan-2026**
COI

Thank you for the thorough revision of the manuscript and for addressing the comments in a clear and coherent manner. The revised version includes several additional analyses and clarifications that are helpful. However, given the newly presented information, I would like to raise a more general point regarding the conclusions. To me, it is somewhat unclear how “potential for cost-effectiveness” is being defined in this context, and whether this statement is fully supported by the results as currently presented. My concerns are outlined below.

First, having read the referenced network meta-analysis, the evidence for the effects of both CBT and mindfulness is graded as low. In addition, the manuscript itself notes that results for physical activity and mindfulness are based on a limited number of contributing studies. The low certainty of the underlying effectiveness evidence is not clearly reflected or discussed, and these two concerns combined is missing in the interpretation/discussion of the results and thus in the overall conclusions.

Secondly, looking at your added CEACs and CE-planes:

o Only two interventions—group-based physical activity and group-based mindfulness—appear to reach borderline probabilities of cost-effectiveness (around 50%) at commonly cited willingness-to-pay thresholds. In contrast, group-based CBT and all individual-based interventions show very low, in some cases close-to-zero, probabilities of being cost-effective.

o The willingness-to-pay line shown in the cost-effectiveness plane appears very steep, which makes its interpretation unclear. At a threshold of £20,000 per QALY, several simulations currently shown as cost-effective would not be expected to fall below the threshold line.

o Furthermore, several sensitivity analyses indicate negative incremental net monetary benefit for individual CBT.

Taken together, these findings suggest a more nuanced interpretation of the results. In particular, there appears to be (1) low-graded evidence for the effectiveness of CBT combined with a very low probability of cost-effectiveness, (2) low-graded evidence for mindfulness, with limited supporting studies and low probability of cost-effectiveness for the individual-based intervention, and (3) moderately graded evidence for physical activity, some uncertainty regarding longer-term effects, and borderline cost-effectiveness only for the group-based intervention.

In light of this, it may be helpful to reconsider or more carefully qualify the conclusion that all interventions studied have the potential to be cost-effective, and to more explicitly reflect the uncertainty in both the effectiveness and economic evidence.

VERSION 2 - AUTHOR RESPONSE

**Reviewer's comments**	**Authors' responses**
**Reviewer 1**	
Conclusion	
Thank you for the thorough revision of the manuscript and for addressing the comments in a clear and coherent manner. The revised version includes several additional analyses and clarifications that are helpful.<br>However, given the newly presented information, I would like to raise a more general point regarding the conclusions. To me, it is somewhat unclear how "potential for cost-effectiveness" is being defined in this context, and whether this statement is fully supported by the results as currently presented. My concerns are outlined below.	We thank the reviewer for the helpful comments.<br>Our responses to the reviewer's comments are provided below.
First, having read the referenced network meta-analysis, the evidence for the effects of both CBT and mindfulness is graded as low. In addition, the manuscript itself notes that results for physical activity and mindfulness are based on a limited number of contributing studies. The	• We acknowledge the reviewer's suggestions and have expanded the text in the discussion section. A key reason for the strength of evidence being graded as low is because of the strict application of the Cochrane Risk of

low certainty of the underlying effectiveness evidence is not clearly reflected or discussed, and these two concerns combined is missing in the interpretation/discussion of the results and thus in the overall conclusions.	Bias v.2.0 tool, with evidence generally judged to be at high risk of bias due to methodological issues inherent in behavioural interventions such as impossibility of blinding in this situation<br><br>• On page 15, we have added a sentence explaining the strength of clinical effectiveness evidence of physical activity promotion, CBT-Fatigue and mindfulness as reported by Leaviss et al. Additionally, we have also provided detail on the reasons behind the GRADE ratings. The strength of the underlying clinical evidence is now also referenced within the conclusions section as this further supports our conclusion that additional research is required to compare these interventions head-to-head.
Secondly, looking at your added CEACs and CE-planes:<br>• Only two interventions—group-based physical activity and group-based mindfulness—appear to reach borderline probabilities of cost-effectiveness (around 50%) at commonly cited willingness-to-pay thresholds. In contrast, group-based CBT and all individual-based interventions show very low, in some cases close-to-zero, probabilities of being cost-effective.	• We have made it clearer on page 11 to explain that the multiway CEAC was generated to assess the probability that each intervention is cost-effective compared with all competing interventions and usual care. As shown in Table 1, the probabilistic INMB values of both group-based physical activity promotion and group-based mindfulness are similar, suggesting comparable cost-effectiveness. As a result, each intervention has an approximately 50% probability of being cost-effective at a willingness-to-pay threshold of £20,000 per QALY gained.

• The willingness-to-pay line shown in the cost-effectiveness plane appears very steep, which makes its interpretation unclear. At a threshold of £20,000 per QALY, several simulations currently shown as cost-effective would not be expected to fall below the threshold line.<br><br>• Furthermore, several sensitivity analyses indicate negative incremental net monetary benefit for individual CBT.	However, the picture is different when considering each intervention in a pair-wise comparison against usual care. We have now also presented the proportion of PSA simulations which fall under the threshold line (in the northeast quadrant) for each pair-wise comparison against usual care on page 11. Except for the CBT-Fatigue (individual) intervention, the probabilities of the remaining interventions being cost-effective when compared to usual care are above 95% at the willingness-to-pay threshold of £20,000 per QALY gained. For the CBT-Fatigue (individual) intervention, the probability is around 63%. However, we accept that CBT-Fatigue has a low probability of being the most cost-effective intervention in the multiway comparison and have highlighted this in the updated conclusions.<br><br>• We apologise for this error. The threshold lines have been amended to accurately reflect the willingness-to-pay threshold of £20,000 per QALY gained.<br><br>• We agree with the reviewer that the cost-effectiveness results of individual CBT-Fatigue is sensitive to several assumptions. As discussed in “resource use and costs” and “cost-effectiveness” sections, individual CBT-Fatigue has the highest costs in the base case due to higher staff contact time resulting in the lowest INMB.

	This was already flagged within the discussion section.
Taken together, these findings suggest a more nuanced interpretation of the results.<br>In particular, there appears to be<br>(1) low-graded evidence for the effectiveness of CBT combined with a very low probability of cost-effectiveness,<br>(2) low-graded evidence for mindfulness, with limited supporting studies and low probability of cost-effectiveness for the individual-based intervention, and<br>(3) moderately graded evidence for physical activity, some uncertainty regarding longer-term effects, and borderline cost-effectiveness only for the group-based intervention.<br>In light of this, it may be helpful to reconsider or more carefully qualify the conclusion that all interventions studied have the potential to be cost-effective, and to more explicitly reflect the uncertainty in both the effectiveness and economic evidence.	We hope that our earlier responses regarding the interpretation of the multiway CEAC and the additional information on the probability of cost-effectiveness versus usual care from the pair-wise comparisons addresses the reviewer's concern about the probability of cost-effectiveness appearing not to support the conclusions.<br>We agree with the reviewer regarding the strength of the clinical effectiveness evidence for all three types of interventions, and have amended the text in the conclusion on page 16. Due to these uncertainties in the underlying evidence, our main conclusion is that further research should be conducted to assess the comparative cost-effectiveness of these interventions which reflects the uncertainty in our findings. We hope our edits to the conclusion section in both the main text and abstract make this position clearer.

VERSION 3 - REVIEW

Reviewer 1

Name Nystrand, Camilla

Affiliation Karolinska Institute, Department of Learning, Informatics, Management and Ethics

Date 26-Mar-2026

COI

No further remarks - excellent work on the revisons!
